# Supplementary material for: Differences in gait parameters between children with achondroplasia and an age-matched control group of typically developed children in the age range of 6 to 12 years
Source: PLoS One. 2025 Jun 18;20(6):e0326184. doi: 10.1371/journal.pone.0326184 (PMC12176110; doi:10.1371/journal.pone.0326184)
Supplement: Attachment — Submitted filename: Achondroplasia reviewer1.docx [file pone.0326184.s005.docx]

The manuscript is acceptable for me after the following revision:

The phrase “Error! Reference source not found.).” appears in two spots in the introduction. Please solve the reason:

- 30 children (15 ACH & 15 CAH) in the age range of 6 to 12 years participated in this study (Error! Reference source not found.).
- Data collection took place at the sports department of the authors university using a 13-camera Vicon System with a sample frequency of 200 Hz. Age (years), height (cm), body mass (kg), sitting height (cm), BMI-SDS and foot-leg- and standing-sitting ratio were recorded (Error! Reference source not found.).

And there are still some language errors. You can use the table below to correct the mistakes:

Language Corrections for Manuscript

| Original | Corrected |
| --- | --- |
| ...occurs in every 25.-30.000 births... | …occurs in approximately 1 in 25,000–30,000 births… |
| ...desired to be narrowed... | ...aimed to narrow the broad age range... |
| ...the consent of participants were taken written... | ...written informed consent was obtained... |
| ...hypothesized to might be caused... | ...hypothesized to be caused... |
| ACH children are under regular care... | Children with ACH were under regular care... |
| ...are causing unwanted load on the joints... | ...cause unwanted loading on the joints... |
| ...at their self-selected walking speed. | ...at a self-selected walking speed. |
| It was desired to get three valid kinetic trials... | Three valid kinetic trials were targeted... |
| ...spatio-temporal, kinematic and kinetic parameters... | ...spatio-temporal, kinematic, and kinetic parameters... |
| ...had significant differences, except the parameter cadence. | ...differed significantly, except for cadence. |
| ...which are decisive for non-significant but great differences. | ...which likely contributed to non-significant findings despite notable mean differences. |
| ...at specific points of the gait cycle gait deviations that are related to the foot-leg ratio. | ...at specific points of the gait cycle, deviations related to the foot-leg ratio were observed. |
| ...a tendency to a greater abduction movement... | ...a tendency toward greater hip abduction... |
| ...not easy to access this cohort and motivate them to join for additional studies. | ...challenging to recruit this cohort for additional studies. |
